# Shiny metal catalysts at the origin of metabolism

**DOI:** 10.1016/j.tim.2026.07.012

**Published:** 2026-08-26

**Authors:** William F. Martin, Nadja K. Hoffmann, Manon L. Schlikker

**Affiliations:** 1 Institute of Molecular Evolution, University of Düsseldorf, 40225 Düsseldorf, Germany

## Abstract

Metabolism is the reaction network that converts inanimate matter into living cells. There is no life without it. The network of approximately 400 enzymatic reactions that synthesize amino acids, nucleosides, and cofactors from compounds in the environment is conserved across life. Before enzymes, how did metabolism arise? The subsurface chemical environment of Earth’s crust reveals clues. Serpentinizing (H_2_ producing) hydrothermal vents contain naturally deposited, shiny metals in their reduced (native) state and phosphite, a reduced form of phosphate. In the laboratory, native metal catalysts fix CO_2_, replacing hydrogenases, the acetyl-CoA pathway to pyruvate, the Krebs cycle, amino acid syntheses, NADH, and ferredoxin. Native metals also phosphorylate organic compounds in water using phosphite, uncovering a new source of energy at the origin of metabolism.

## Metabolism arose at a time and place

We know that life arose once because all life uses the same genetic code. We also know roughly when life arose because 3.4 Ga (billion year old) hydrothermal vent settings harbor microbial microfossil communities [1,2], some bearing isotope signatures of the acetyl–CoA pathway (Wood–Ljungdahl pathway) (see Glossary) [3], the most ancient pathway of CO_2_ fixation and the only one that occurs in both bacteria and archaea [4]. Autotrophic metabolism was thus off and running by 3.4 Ga at the latest. How did it start?

Theories on how metabolism started are inseparable from the question of where metabolism started, because the environment has to provide carbon, nitrogen, and phosphorus as starting materials for organic synthesis, a constant supply of energy, and catalysts that can accelerate slow reactions. Metabolism is a set of exergonic chemical reactions powered by energy gleaned from the environment. Chemical reactions only go forward if they release energy, but they often require catalysts to overcome the hurdle of activation energy. Although all reactions can take place without catalysts, the uncatalyzed rate is usually too low to be relevant for metabolism or origins—some uncatalyzed reactions have a half-life of more than 2 billion years, with enzymes increasing reaction rates by up to 10^18^ fold [5]. Questions concerning the origin of metabolism entail organic synthesis, energy, primordial environmental catalysts, and the end product of metabolic evolution: modern biosynthetic pathways.

If we look for the simplest and most primitive energy-harnessing reactions among modern cells, acetogens (bacteria) and methanogens (archaea) have always stood out [4,6–9]. These chemolithoautotrophs have rock bottom nutrient requirements, growing from H_2_, CO_2_, NH_3_, H_2_S, and mineral salts. They harness energy to produce acetate (acetogens) or methane (methanogens). Both groups harness energy from the reduction of CO_2_ with H_2_ involving reacions of the acetyl–CoA pathway: in acetogens to make acetate and in methanogens to make the methyl group leading to methane synthesis. The acetyl–CoA pathway is the only pathway of coupled carbon and energy metabolism known, and the only CO_2_ fixing pathway that traces to the last universal common ancestor (LUCA) [10]. Some modern hydrothermal environments produce methane from H_2_ and CO_2_ through abiotic reactions [11], possible geochemical precursors of the first biochemical reactions [12]. Acetogens and methanogens use the acetyl–CoA pathway to synthesize pyruvate, the central biosynthetic intermediate [6]. In acetogens, this requires an input of one ATP [4], which is however recovered via chemiosmotic coupling, while in methanogens pyruvate synthesis via the acetyl–CoA pathway is powered by redox reactions and requires no ATP input. Both groups couple exergonic H_2_-dependent CO_2_ reduction to ion pumping at the plasma membrane for chemiosmotic ATP synthesis [13]. Acetogens and methanogens furthermore synthesize all of their organic compounds from H_2_, CO_2_, NH_3_, H_2_S, and inorganic phosphorus. Therefore, their enzymatic reaction networks provide a living catalog of ancient metabolism, an outline of all 420 reactions that life needs to synthesize amino acids, bases, and cofactors from gases and salts present on the early Earth [14].

Carbon flows generously through the carbon metabolism of chemolithoautotrophic cells. For every carbon atom in CO_2_ that is fixed as cell mass, approximately 20 carbon atoms exit the cell as acetate (or methane) during growth [6]. In energy metabolism, by contrast, phosphate is greedily sequestered: *Escherichia coli* synthesizes and hydrolyzes approximately 100 ATP for every phosphate residue that ends up in cell mass [15]. Primordial metabolism can only have been less efficient in its use of phosphate bonds as an energy currency, which highlights the need for a constant environmental supply of energy and phosphorus to get metabolism started at the origin of life. What kind of environment can deliver those supplies?

### Environments for origins: energy and phosphate

All theories for metabolic origin require a source of energy to power the first chemical reactions of life before cells or enzymes existed. Those sources of energy are diverse, as summarized in Table 1. Some theories derive the energy for metabolic origin from organic compounds present in the environment, but the source of those organic compounds is actually the issue. Organics from meteorites are of no use for metabolic origin, as they are unfermentable substrates—too reduced to support disproportionation (fermentation) reactions as an energy supply [17]. Theories that envisage the origin of metabolism in the atmosphere or on the Earth’s surface tend to rely on ultraviolet (UV) light, sometimes with the help of meteorite impacts to provide reactive forms of phosphorus (Box 1). As a source of energy, UV light breaks bonds in water molecules, generating H• and HO• radicals that rapidly react with carbon compounds, inducing bond cleavage and formation reactions [22]. However, UV-induced organic reactions typically involve no catalysts, nor do any cells obtain energy from UV light. This leaves no discernible path of continuity [42] between UV-induced reactions and microbial metabolism, the reactions of which are catalyzed by enzymes. The use of mass spectrometry (MS) to record product formation in origins studies (Box 1) has the caveat that organic synthesis reactions also take place in the sprayed microdroplets produced by mass spectrometers [43]. Microdroplet chemistry has become an established method of organic synthesis [44], also in origins studies [45,46]. The mechanism is interesting and was long overlooked. Microdroplets bear an electrostatic charge on their surfaces, orienting charged reactants (most metabolites are) along the field, bringing them into position for reaction. Substrate orientation along electrostatic fields in enzyme active sites is now recognized as a widespread mechanism of enzymatic catalysis [47].

In autotrophic theories of metabolic origin, metabolism starts with CO_2_ as in all modern ecosystems, and the energy for metabolic origin stems from light-independent chemical reactions that take place in the dark confines of volcanic environments or submarine hydrothermal vents [48] (Box 2).

Having a source of energy at the origin of metabolism is one thing. Having a means to conserve energy is quite another. Microbes have no mechanisms for conserving energy supplied by UV light or by bolide impacts. Metabolism conserves chemical energy, mainly using phosphate. In energy metabolism, a portion of the energy released during exergonic reactions is conserved in the form of high-energy phosphate bonds [49]. The hydrolysis of those bonds releases energy (Δ*G*_*0*_′ = −32 kJ·mol^−1^ in the case of ATP), which can be coupled to otherwise endergonic reactions, allowing them to go forward. Environmental sources of phosphate and ATP pose high hurdles for theories of metabolic origin because of what is known as the ‘phosphate problem’of early metabolic evolution [50,51], which has two facets: (i) phosphate is poorly soluble in water, which results in low bioavailability at the origin of life, and (ii) it is very unreactive, rendering it chemically challenging to incorporate phosphate into prebiotic syntheses [28,52,53]. Two of Westheimer’s classical [49] questions have remained unanswered: where did phosphorylating compounds come from on a barren Earth, and how were they incorporated into the conserved metabolic network of life?

Box 1 **Origin of metabolism in surface environments**

Starting with Haldane’s concept of life arising from fermentations in an ocean (Table 1A) consisting of organic soup [16], all theories for origins are set in a specific environment, because the environment impacts the kinds of molecules, reactions and processes that can occur. Today we know that fermentations cannot have been the starting point of metabolism [17]. Nonenzymatic glycolytic reactions from glucose-6-phosphate to lactate were reported in 1967 [18]. Reactions of the pentose phosphate cycle also occur in water without enzymes [19], but this does not explain where sugars and sugar phosphates come from. An organosulfidic model (Table 1B) [20,21] posits that metabolism arose in a sulfide-rich environment that contained all organics found in cells, a compound set called the KEGG (Kyoto Encyclopedia of Genes and Genomes) ‘universal’ metabolism set (https://www.genome.jp/kegg/). The organosulfidic model excludes, however, compounds containing phosphate, positing that thioesters are the ancestral energy currency in early chemical evolution. The model does not explain where environmental thioesters come from or address how phosphate entered metabolism. Atmospheric chemistry is the source of metabolism in some models (Table 1C), with UV light serving as the energy supply to activate CO_2_ for synthesis of organics in the gas phase. Ueno *et al*. [22] recently reported UV-dependent synthesis of an impressive spectrum of relevant organic compounds from CO_2_ and H_2_O, as revealed by MS (see main text). In a terrestrial surface model that is very popular among chemists (Table 1D), the energy for organic synthesis comes from meteorite impacts [23,24]. Meteorite impacts release massive energy, which can theoretically synthesize cyanide from atmospheric gases [24], but against cyanide’s very steep free energy of formation of +125 kJ·mol^−1^ [25]. The energy in cyanide supports synthesis of amino acids [26], bases [23], and cofactors [27] in those models. Phosphorylation in the impactor model is supplied by schreibersite (Ni_*n*_Fe)P [23,24], a highly reactive phosphide mineral naturally found only in meteorites [28]. Prebiotic amino acids as a source of cyanide have also been suggested [29]. In a terrestrial hot springs model (Table 1E), UV light reacts with ZnS [30] to provide energy and electrons, with organics from cyanide chemistry as above, but with the energy for phosphorylation stemming from wet–dry cycles [31]. A mixed energy proposal obtained methanol from gas phase CO_2_ interacting with H_2_ on FeS minerals with energy from UV light [32]. Models A and B do not address nitrogen in metabolism, and models A–E do not evoke a major role for catalysts at metabolic origin.

Box 2 **Autotrophic theories posit a subsurface origin within the crust.**

The volcanic and hydrothermal vent theories (Box 1) are designed along the lines of microbial metabolism and start from CO_2_, like metabolism in autotrophs. Both derive their energy from chemical reactions that take place within the Earth’s crust, as opposed to requiring UV light or meteorite impactors. Wächtershäuser’s proposal (Table 1F) starts from a hypothetical version of the reverse tricarboxzlic acid (rTCA) cycle in which thioacids (R–COSH) preceded organic acids (R–COOH) in biochemical evolution, with the energy and electrons to drive those reactions stemming from pyrite (FeS_2_) synthesis [33], FeS + H_2_S → FeS_2_ + 2e^−^ + 2H^+^. Pyrite synthesis is, however, energetically less favorable than originally thought [34]. Pyrite is very stable, but methanogens can reductively degrade it to obtain iron [35] and can even grow on H_2_ produced by pyrite synthesis [36]. FeS catalysis is a cornerstone of the ‘FeS world’ theory [37], with substrate binding to FeS surfaces bringing substrates into proximity for reactions. But FeS would not reduce CO_2_, hence something was missing in the theory. The argument favoring FeS as a reductant for CO_2_ fixation is based on the midpoint potential of H_2_ → 2e^−^ + 2H^+^ (*E*_0_′ = −414 mV) [33], which is not sufficient for CO_2_ fixation. But serpentinizing hydrothermal vents are very alkaline, pulling the H_2_ oxidation reaction to the right, such that midpoint potentials exceeding −800 mV are reached in nature [7,38]. As a consequence, CO_2_ is reduced by H_2_ to formate and methane via abiotic reactions in modern serpentinizing vents [8,9,11]. The hydrothermal vent theory (Table 1F) is built on the acetyl–CoA pathway and H_2_ from serpentinization [39]. Varma *et al*. [40] showed that Fe^0^ efficiently reduces CO_2_ to formate and acetate. Preiner *et al*. [41] found that awaruite, Ni_3_Fe, a common naturally occurring alloy in serpentinizing hydrothermal vents, catalyzes H_2_-dependent CO_2_ reduction to formate, acetate and pyruvate, functionally replacing the ten enzymes and 15 cofactors of the acetyl–CoA pathway. Subsequent work showed that native metals germane to hydrothermal vents (Fe^0^, Ni^0^, Co^0^, and Pd^0^) would catalyze reactions of the TCA cycle, amino acid synthesis, and even phosphorylation. In the serpentinizing vent theory for the origin of metabolism, native metals in their shiny, elemental state are the decisive catalysts (Table 1G) and the first forms of life to emerge at origins are acetogens and methanogens [39], which live from H_2_ and CO_2_ (or formate) and thrive in serpentinizing systems today [8,9]. Other theories for metabolic origin are silent on the physiology of the first cells.

### What is metabolism?

In the context of early evolution, metabolism can be meaningfully outlined as the reactions that bacteria and archaea use to synthesize 20 amino acids, the eight (unmodified) bases of RNA and DNA, and approximately 18 cofactors from maximally simple substrates: H_2_, CO_2_, NH_3_, H_2_O, H_2_S, and P_i_ (inorganic phosphate). Those 420 reactions [14,54] produce the compounds required to make proteins from the information in genes, provided that there is a source of ATP. Those reactions represent core metabolism. The reactions are as universal as the genetic code or ATP synthases, notwithstanding differences in biosynthetic pathways across bacteria and archaea [14,55,56], and they are broadly exergonic in the biosynthetic direction [54]. Notably, the 420 reactions themselves are more highly conserved across bacteria and archaea than the enzymes that catalyze them. Only about half of the enzymes catalyzing core metabolic reactions trace back to LUCA [14]. This indicates that a functioning organic reaction network, catalyzed by the environment, existed before the full set of metabolic enzymes had arisen in the bacterial and archaeal lineages, pointing to the existence of autocatalytic networks at the onset of metabolism [57,58].

**Figure 1:**
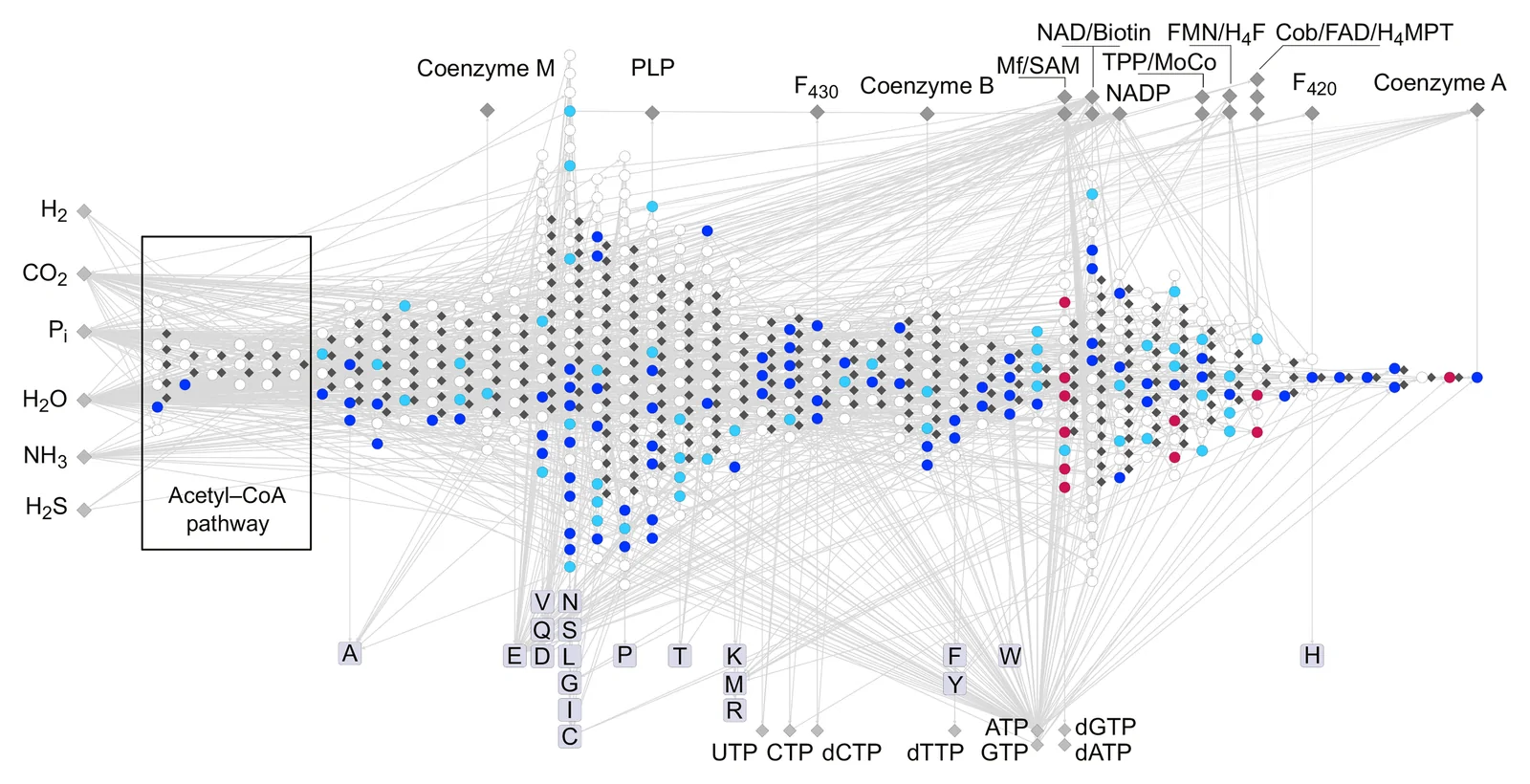
A vectorial representation of metabolism highlighting the role of phosphate. Diamonds (compounds) are connected by reactions (circles). The starting compounds of metabolism are indicated on the left (see text). The network of 420 reactions is redrawn from reference [14]. All 149 reactions (35%) involving phosphate bonds are color-coded as follows: red circles represent the 11 reactions in which nucleotides (ATP, ADP, or GTP) participate directly as substrates rather than solely as energy carriers, for example, adenylylation in NAD or FAD synthesis. Dark blue circles denote 83 reactions (20%) energetically driven by phosphate bond cleavage (e. g., coupled to ATP hydrolysis). Light blue circles indicate 55 reactions that otherwise involve formation or cleavage of phosphate bonds. There are 142 additional reactions in core metabolism involving phosphorylated substrates or cofactors in which phosphate bonds are not affected, bringing the total to 291 out of 420 reactions in core metabolism (69%) that involve phosphate [14]. For comparison, 32 reactions in core metabolism (7.6%) [14] involve thioesters [20]. The apparently small number of reactions involving PLP is a result of KEGG (Kyoto Encyclopedia of Genes and Genomes) coding, in which cofactors that leave the reaction unchanged (as in transaminations) are not listed as participants in the reaction because the same compound appears on both sides of the reaction. The box on the left indicates reactions of the acetyl–CoA pathway to pyruvate (the single diamond in stratum 7) plus carbamoyl phosphate synthase (blue dot in stratum 2). The blue dot in stratum 3 is bacterial formyl-tetrahydrofolate synthase. CoA: coenzyme A; Cob: cobalamin (vitamin B_12_ or related cobamides); FAD: flavin adenine dinucleotide; FMN: flavin mononucleotide; H_4_MPT: tetrahydromethanopterin; Mf: methylfactor; MoCo: molybdenum cofactor; PLP: pyridoxal 5′-phosphate; SAM: S-adenosylmethionine; TPP: thiamine pyrophosphate.

The set of 420 reactions in H_2_-dependent chemolithoautotrophic metabolism is shown in Figure 1 (Key figure), in which the reactions have been ordered according to the availability of reactants, a procedure called stratification, developed by Mike Steel and Daniel Huson [14]. In stratum (layer) 1 (on the far left of Figure 1) are the starting materials provided by the environment: gases and P_i_. Instratum 2 are all compounds that are made by enzymes using only compounds present in stratum 1. In stratum 3 are all compounds that can be enzymatically synthesized only from those compounds in strata 1 and 2, etc., repeating until all compounds of metabolism are produced. Starting from environmental gases, amino acids are synthesized first, nucleoside triphosphates next, and cofactors last. It is not known whether the ordered sequence of reactions in core metabolism recapitulates the order of their emergence, but it is a possibility. Note that many cofactors are required for reactions that precede their synthesis (Figure 1), indicating that environmental catalysts must have served in their stead before coenzymes were available, which is the topic of the next section.

### Carbon chemistry, the strong suit of native metal catalysts

The first problem nature had to solve on the path to the origin of metabolism was obtaining organic compounds from CO_2_. That requires CO_2_—the main carbon species on early Earth [59]—and a continuous supply of reductant to fix CO_2_. Chemolithoautotrophs use H_2_ as their electron source, which is produced by serpentinization (see below). The first seven strata in Figure 1 contain the acetyl–CoA pathway of methanogens and acetogens, reactions that convert H_2_ and CO_2_ to pyruvate. In cells, those reactions require 15 enzymes and nine cofactors: the molybdenum cofactor (MoCo) for formate synthesis, tetrahydrofolate (H_4_F) or tetrahydromethanopterin (H_4_MPT) as C1 carriers, NADH and F_420_ (an archaeal variant of FADH) for hydride transfers, reduced ferredoxin (Fd^−^) for one-electron transfers, methanofuran and ATP for formyl transfer, cobamide (Cob, a form of cobalamin) for methyl transfer, thiamine pyrophosphate (TPP) for C2 transfer at pyruvate synthase [60], in addition to NiFeS centers in acetyl–CoA synthase (ACS) and carbon monoxide dehydrogenase (CODH) for formation of carbon–metal bonds, and NiFeS centers in hydrogenase active sites to extract electrons from H_2_ [61–63]. How were those reactions possible before there were enzymes or cofactors? Shiny native metals that naturally occur in serpentinizing systems can replace the entire acetyl–CoA pathway and many other enzymatic reactions of metabolism (Table 2).

Serpentinizing hydrothermal vents (see below) contain native metals, including Ni^0^, Fe^0^, and alloys such as Ni_3_Fe (awaruite), in addition to native cobalt and platinum-group elements (PGEs) [90,91] and they contain vast amounts of continuously synthesized H_2_. A number of studies have now shown that Ni^0^ alone (or Ni_3_Fe alone) can act as a single, broad-specificity catalyst to replace all enzymes and cofactors of the acetyl–CoA pathway, synthesizing pyruvate from H_2_ and CO_2_ overnight at 50–100°C in water: up to 10 μM after 16 h at 100°C with Ni_3_Fe [41], up to 20 μM after 24 h at 25°C with Ni_3_Fe [68], and up to 100 μM after 24 h at 100°C with either Ni^0^ or Ni_3_Fe [70]. With Fe^0^ there is no need to add H_2_ [40]. A list of reactions relevant to the origin of metabolism that are catalyzed by native metals under hydrothermal vent conditions is given in Table 2. In those reactions, H_2_-dependent reduction of CO_2_ is the source of energy, just as in acetogenic and methanogenic metabolism, and the native metals are catalysts that replace both enzymes and cofactors.

The metals that perform CO_2_-reducing catalysis in the laboratory (Table 2) perform functions in the acetyl–CoA pathway. Nickel is a particularly important catalyst in early biochemical evolution as a catalytic atom in three enzymes of the acetyl–CoA pathway (FeNi hydrogenase, CODH, and ACS) and as a component of two cofactors: the tetrapyrrole F_430_ in methanogenesis and the nickel pincer nucleotide cofactor of racemases [92]; catalysis by Ni^0^ in water can replace a surprisingly broad spectrum of enzymatic reactions [83]. Iron is present in FeS clusters that coordinate Ni in the acetyl–CoA pathway [61–63]. Cobalt is present in cobamide (a form of cobalamin), where it binds a methyl group for transfer to a Ni atom at the ACS active site [93]. Molybdenum (or tungsten) is required in the MoCo for the conversion of CO_2_ to formate [94]. Its sulfide, MoS_2,_ catalyzes an impressive spectrum of organic reactions at high pressure and 250°C, converting H_2_ and CO_2_ to intermediates of numerous CO_2_-fixing pathways [95].

The CO_2_-reducing reactions catalyzed by native metals produce large amounts of products: up to 0.33 M formate (Ni_3_Fe, 100°C, 16 h) [41], 800 μM acetate (Ni^0^, 100°C, 168 h) [70], and even up to 200 μM pyruvate (Ni^0^, 100°C, 168 h) [70], equaling the cytosolic concentration of pyruvate in growing acetogen cells [96]. Ni^0^ can also catalyze reactions of the reverse citric acid cycle [78], reductive aminations in amino acid synthesis [78,79], citramalate synthesis [68], reactions of sugar metabolism (which require almost no catalysis [18,19]), carbonyl and vinyl reductions [83], NAD^+^ reduction [64,65], and pyridoxal amination [79]. Native cobalt is also an effective catalyst for CO_2_ reduction to prebiotic organics [69]. At higher hydrothermal pressures and temperatures, Co^0^ can also convert H_2_ and CO_2_ to hydrocarbons up to C24 in length [75]. There are reports of FeS or FeNiS catalysts generating acetate or pyruvate, but these involve externally applied electrochemical voltages on FeNiS electrodes [97], whereby the metal sulfides on the electrodes are reduced during the experiment to native metals [98], which are the active catalysts. A recent study highlighted the surprising ubiquity of native metal catalysis. Boé *et al*. [72] reported the synthesis of formate, acetate, methanol, CO and methane from H_2_ and CO_2_ in the absence of any added catalysts—the interior walls of the reactor, made of a shiny FeNiCr alloy, were catalyzing the reactions [72]. Many continuous-flow reactors have been built that simulate aspects of hydrothermal vents [99], but none have been operated so far with H_2_ and native metal catalysts added (Table 2).

In a geochemical context, the H_2_ required by many reactions in Table 2 can be supplied by serpentinization. Serpentinization is a geochemical process that produces H_2_ and alkaline fluids in the effluent of some hydrothermal vents through chemical reactions of Fe(II)-rich rocks with water. Serpentinization has been going on for as long as liquid water has existed on Earth [100,101]. The substrates for serpentinization are **ultramafic** rocks. Rocks with high magnesium and iron content are called mafic (from magnesium- and ferrum-rich), whereas those with very high Mg^2+^ and Fe^2+^ content are called ultramafic. During serpentinization, Fe(II) in mafic rock is oxidized to Fe(III), and water is reduced to H_2_, with the oxygen in water forming the mixed-valence iron oxide magnetite (Fe_3_O_4_). Chamberlain *et al*. [90] summarize the reaction as

6Mg_1.5_Fe_0_._5_SiO_4_(olivine) +7H_2_O → 3Mg_3_Si_2_O_5_(serpentine) + Fe_3_O_4_(magnetite) + H_2_

while Sleep *et al*. [100] simplify the reaction to its basics:

3FeO (in rock) + H_2_O (liquid) → Fe_3_O_4_ (magnetite) + H_2_ (in solution)

Serpentinizing hydrothermal systems emit H_2_ in their effluents at concentrations of up to 5 mmol per liter [12], orders of magnitude more than methanogens and acetogens require to grow [102, 103]. The two simplified reactions given above omit an important component, namely the formation of brucite, Mg(OH)_2_, which makes the effluent of serpentinizing systems alkaline [104], with a pH in the range of 8–13 [12]. Alkalinity renders the H_2_-containing effluent extremely reducing, because OH^−^ ions consume protons to form water, which pulls the H_2_ oxidation reaction, H_2_ → 2H^+^ + 2e^−^ to the right. As a result, the midpoint potential of H_2_ oxidation in the effluent of serpentinizing systems is not −414 mV (as for pH 7) but on the order of −800 mV [7,38], sufficient to reduce transition metals (Fe^2+^, Ni^2+^, and Co^2+^) to their zero-valent (native) state (Fe^0^, Ni^0^, Co^0^,and their alloys) [90]. These metals are effective catalysts for CO_2_ reduction, which is why the effluent of serpentinizing systems often contains abiotically generated formate and methane [9]. Magnetite, a direct product of serpentinization, is also an effective catalyst for H_2_-dependent CO_2_ reduction to prebiotic organics [41], and is the main catalyst in the Haber–Bosch process for N_2_ reduction to NH_3_. Newer reports indicate that serpentinization reduces large amounts of N_2_ to NH_3_ in modern seafloor environments [105]. Serpentinizing systems harbor native metals [90,91] and phosphite [106] because of their highly reducing environments.

### A surprising turn in the phosphate problem

The reactions of the acetyl–CoA pathway entail only one ATP-dependent step, the formyltetrahydrofolate synthase reaction of acetogens (second blue dot from the left in Figure 1). Where does the energy come from to drive pyruvate synthesis from H_2_ and CO_2_ in the acetyl–CoA pathway and in its Ni^0^-catalyzed analog reactions [41,70]? It comes from redox chemistry: in the absence of strong oxidants (such as O_2_), the equilibrium in the reaction of H_2_ with CO_2_ lies on the side of reduced carbon compounds. But H_2_ and CO_2_ are both generally inert; they have to be activated to react. In stratum 1 of core metabolism are four enzymes with Fe(Ni)S centers: two that activate CO_2_ by forming carbon–nickel bonds (CODH and ACS) [61–63] and two hydrogenases that activate H_2_ by forming Ni- or Fe-hydrides [65]. The shiny native metals themselves perform the same activation function as the enzymes (Table 2). The subsequent reactions to pyruvate are energetically downhill in metabolism [4] and occur spontaneously in water on native metal surfaces [41]. Of course, native metals have a century-long tradition as versatile catalysts in organic chemistry [107]. Their occurrence in H_2_-producing hydrothermal vents [90,91] and their ability to replace enzymes and cofactors [14] now bring that multifaceted chemistry into an origin-of-metabolism context. Cells reduce ferredoxin, the main carrier of electrons in the cytosol of acetogens and methanogens, with H_2_ [66]. Reduced ferredoxin is a metabolic energy currency in its own right, in addition to ATP [108, 109]. H_2_ from serpentinization can account for a substantial part of the environmental energy; what about phosphate?

Out of 420 reactions in Figure 1, 149 (35%) involve the formation or breakage of phosphate bonds; they are color-coded in the figure. Among those 149 reactions, 94 (22% of all metabolic reactions) involve hydrolysis of a phosphoanhydride bond in ATP, ADP, GTP, GDP, or CTP (color-coded dark blue and dark red in the figure). They are uniformly distributed across metabolism. The 11 red circles (2.6% of the total) designate a subset of the 94 NTP-hydrolyzing reactions in which a nucleotide moiety (usually AMP) is attached to a biosynthetic intermediate, usually a cofactor, as in NAD^+^, FAD, coenzyme A, or similar. The 83 dark blue circles designate reactions in which phosphoanhydride bonds are hydrolyzed without adenylation. Of those, 33 represent classical textbook examples of bioenergetic coupling, in which ATP (or GTP) hydrolysis is enzymatically coupled to the reaction, contributing −32 kJ·mol^−1^ to its energetics under physiological conditions so that it will go forward. The other 50 are ATP- or GDP-dependent phosphorylations that activate moieties in substrates for further reaction. The remaining 55 light blue circles indicate reactions involving phosphate bonds but not phosphoanhydrides, such as phosphatases or other phosphate-producing reactions. Clearly, phosphate is hard-wired into the fabric of metabolism. The metabolic network of the first cells cannot have arisen without the participation of high-energy phosphate bonds. Where did those high-energy phosphate bonds come from?

This has been a key question in metabolic evolution for as long as anyone can remember: How did phosphate come to assume such a central role in bioenergetics? The classical answer from origin-of-life chemistry has been that highly reactive forms of phosphate, such as diamidophosphate [52], phosphides [23], or P_2_O_5_ [53] were somehow present in ancient environments, or that cyanide-derived condensing agents such as cyanoacetylene, cyanogen, cyanamide, or cyanate were available in ancient environments [52], or that wet–dry cycles simulated by recurrent oven drying in the laboratory [31,53] were the source of phosphorylated organics. While those routes are, in principle, possible, they offer no evolutionary transition to enzymatic phosphorylation, because enzymes will not survive harsh conditions such as aggressive chemicals or drying [15]. Insights from microbial physiology and serpentinization open up new perspectives on the source of phosphate at the origin of metabolism (and life).

### Phosphite and native metals phosphorylate

A number of microbes are known that can obtain all of their phosphate and, in some cases, all of their electrons from phosphite $\left({\text{HPO}}_{3}^{2-}\right)$, which must be oxidized to phosphate for metabolism [110–112]. Mao *et al*. [113] recently characterized the phosphite-oxidizing enzyme, AMP-dependent phosphite dehydrogenase (AdpA), from *Desulfotignum phosphitoxidans* (Gram−) and *Phosphitispora fastidiosa* (Gram+). It catalyzes the reaction ${\text{HPO}}_{3}^{2-}+\text{AMP}+{\text{NAD}}^{+}\to \;\text{ADP}\;+\;\text{NADH}\;$, which is a rare case of a redox reaction performing substrate-level phosphorylation, as bacteria can readily interconvert two ADP into ATP and AMP via adenylate kinase. The reaction itself is interesting because it is a one-enzyme system of oxidative phosphorylation, as in mitochondria, except that phosphite rather than the acetyl moiety of acetyl–CoA is oxidized and no ion gradient or ATPase is required. The environmental source of phosphite is of interest. Bacteria that inhabit serpentinizing vents exhibit conspicuously high frequencies of phosphite-oxidizing genes [12,114,115] indicating that they are using phosphite present in those environments. Moreover, Pasek and colleagues have shown that some serpentinizing systems also contain phosphite [106], in line with the frequency of phosphite-oxidizing genes in microbes living in such environments. The phosphite oxidation reaction is highly exergonic: ${\text{HPO}}_{3}^{2-}+{\text{H}}_{2}\text{O}\to {\text{HPO}}_{4}^{2-}+{\text{H}}_{2}$ with Δ*G*_*0*_ ′ = −46 kJ·mol^−1^ [111], yet phosphite oxidation does not take place spontaneously in water; the reaction requires a catalyst.

Yang and Metcalf [111] showed that bacterial alkaline phosphatase catalyzes the H_2_-producing phosphite oxidation reaction. Can shiny (native) metals that occur naturally in serpentinizing systems also catalyze phosphite oxidation in water? Awaruite (Ni_3_Fe) and Ni^0^ can, but native palladium (Pd^0^) works better [14,86]. Like Ni^0^, native palladium occurs in serpentinizing systems [91]. Native metals are proficient at replacing enzymes and cofactors (Table 2). Can they substitute for both the enzyme and NAD^+^ in the AdpA reaction? In water, Pd^0^ catalyzes the reaction ${\text{HPO}}_{3}^{2-}+\text{AMP}\to \text{ADP}+{\text{H}}_{2}$ [14,86] which is exergonic with Δ*G*_*0*_′ = −15 kJ·mol^−1^ (Table 3).That is, Pd^0^ replaces AdpA, with protons from water substituting for NAD^+^ as the oxidant. It might seem odd that water serves as the oxidant, but phosphite is a very strong reductant, with *E*_*0*_′ = −690 mV [110]. Further experiments showed that Pd^0^ catalyzes a broad spectrum of phosphite-dependent phosphorylation reactions [86] (Table 3), including the phosphorylation of acetate (a common product of metal-catalyzed CO_2_ fixation) to generate acetyl phosphate, which itself is a potent phosphorylating agent that phosphorylates ADP to ATP in the acetyl–CoA pathway of acetogens [13]. The yields in these reactions are modest from a chemist’s perspective, with 50% conversion in the case of phosphoserine, 8% conversion in the case of acetyl phosphate, and approximately 1% conversion in other cases so far [86]. But the yields are physiologically relevant: they meet or exceed the concentrations of the corresponding phosphorylated metabolites in the cytosol of growing *E. coli* cells [15]. The mechanism of the metal-catalyzed, phosphite-dependent phosphorylation reactions probably involves hydride removal by the metal surface and a metaphosphate [PO_3_]^−^ intermediate [86]. Wolfgang Buckel suggested 25 years ago [117] that phosphite oxidation might have served as a phosphorylating reaction at the origin of metabolism.

**Outstanding questions**

Dozens of flow reactors have been constructed and reported as simulated hydrothermal vent systems, with modest results so far. Will flow reactors generate more diverse product spectra if they employ H_2_ and native metals as catalysts?

Phosphite is utilized by many microbes, but it has been detected in only a handful of hydrothermal systems. More sampling is needed to survey the occurrence of phosphite in modern environments.

Can native metals replace all core metabolic reactions, or are there cases where other novel forms of prebiotic catalysis, for example electrostatic fields in microdroplets, are required?

Many soluble divalent ions, in particular Mg^2+^, play essential roles at the active sites of metabolic enzymes. How do divalent ions impact catalysis by native metals?

A number of studies have focused on the natural ion and redox gradients at hydrothermal vents. Aside from the universally conserved rotor–stator ATP synthase, are there any core metabolic reactions that strictly require ion gradients to go forward?

The mechanisms of reactions catalyzed by native metal catalysts are largely unknown. Mechanistic studies using density functional theory that can help close that gap are lacking.

Investigations of metabolic origin have traditionally evoked FeS catalysis. Sulfur is not required in any of the reactions catalyzed by native metals so far. Was the main function of sulfur at origins not catalytic but structural instead—to coordinate metals in proteins?

Newer studies using native metals uncover a broader product spectrum that includes phosphorylation, but Pd never made it into metabolism. What other elements could have contributed to catalysis at origins. Lanthanides are one example, but are there others?

What other (so far under investigated) catalysts exist in serpentinizing hydrothermal vents?

If Pd^0^ is such an efficient catalyst, why was it never incorporated into metabolism? This issue was recently addressed for the case of native nickel [83]. Nickel catalyzes a broad spectrum of reactions *in vitro* as Ni^0^ nanoparticles (Table 2) and catalyzes many reactions *in vivo* as Ni^2+^ ions that are coordinated in enzymes [61] and cofactors [92] by N, S, O, and C atoms. Cells have sufficient reducing power to convert Ni^2+^ to Ni^0^ (*E*_0_′ = −260 mV) using low-potential ferredoxins (*E*_0_′ = −450 mV). The problem with Ni^0^ is that its substrate specificity is too broad. Cells would be unable to keep cytosolic Ni^0^ from reacting indiscriminately with H_2_, NADH, carbonyl or vinyl moieties, and the like. Such an uncontrollable catalyst would hardly fit within the network of high-specificity enzymatic reactions that comprise metabolism (Figure 1) [83]. The same reasoning applies to Pd^0^, which phosphorylates many substrates using phosphite (Table 3), except that Pd^0^ is far more noble than nickel. Like gold, Pd^0^ resists oxidation, even in the presence of O_2_, posing a seemingly insurmountable challenge for the first cells to generate Pd^2+^ for import into the cytosol. The virtue of native metals at life’s origins—unfettered activity to promote many different reactions—is their flaw in enzymatic metabolism. When metalloenzymes and metallocofactors arose in evolution [14], the ancestors of cells could synthesize catalysts tailored to their needs using soluble metal ions, replacing native metals, thereby freeing metabolism from solid-state catalysts in the crust (Table 1G).

### Concluding remarks and future perspectives

The reaction catalyzed by AdpA is an example of substrate-level phosphorylation; it conserves energy from phosphite oxidation in the phosphoanhydride bond of ADP [113]. Note that the Pd^0^-catalyzed AMP-phosphorylating reaction (Table 3) also conserves energy. The Pd^0^-catalyzed acetate-phosphorylating reaction conserves even more energy (Table 3), because the free energy of hydrolysis of acetyl phosphate, an acyl phosphate, is −43 kJ·mol^−1^ whereas that of ADP to AMP is −31 kJ·mol^−1^. Is this the source of phosphorylation at the origin of metabolism? Moreover, is it also the source of biological energy conservation using phosphate? It is possible. The reactions take place in water, and several different types of metabolically relevant moieties (hydroxyl, carboxyl, amino, and phosphoryl) are phosphorylated. The reactions proceed under conditions found in serpentinizing hydrothermal vents (alkaline, 25–100°C) that also support CO_2_ fixation, TCA cycle reactions, hydrogen activation, and amino acid synthesis (Table 2). This points to a common site and continuity [42] for the concerted origin of carbon and energy metabolism. This would help to explain why phosphorylations are more or less uniformly distributed across metabolism (Figure 1) and interwoven into (bio)synthetic reactions: in a single primordial environment, redox chemistry provided by H_2_ was a geochemical source of energy for CO_2_-reducing biosynthetic reactions, while phosphite oxidation was a geochemical source of energy in metabolic reactions where H_2_ offered no help. Over geological timescales, this kind of energetic synergy between exergonic redox reactions and phosphorylations could, in principle, produce a reaction network resembling the modern state of metabolism shown in Figure 1.

Serpentinizing hydrothermal vents generate natural pH gradients, the effluents being more alkaline than sea water [39]. Once translation and proteins had arisen, these naturally formed pH gradients could have powered the first rotor–stator ATP synthases, provided that a simple fatty acid bilayer was present [118]. However, the reactions summarized in Table 2 do not require a gradient to go forward (see Outstanding questions). They take place in batch reactions at the interface between the aqueous phase and the solid-state catalyst and are thermodynamically driven by redox potentials: the reducing power of H_2_ (*E*_0_′ = −414 mV, but *E*_0_′ = −800 mV at pH 10 [7]), of the metals themselves (*E*_0_′ = −470 mV for Fe^0^ [119], *E*_0_′ = −260 mV for Ni^0^ [79]), or of phosphite (*E*_0_′ = −690 mV [110]), the latter generating H_2_ as a product of oxidative phosphorylation reactions. The reactions catalyzed by native metals furthermore require neither UV light, bolide impacts, iron sulfides, iron oxides, externally applied voltages, electrophilic condensing agents, complicated microfluidic apparatus, nor RNA to go forward, but only substrates in water in contact with solid-state catalysts. This simplicity invites the further study of interactions of cofactors [64,65,79] and proteins [66] with native metal surfaces. Some methanogens and acetogens can grow today on native Fe^0^ as an energy and electron source [120–122]. If metabolism arose with the help of native metals, it follows that enzymes interacted with metal surfaces during the transition from metal-catalyzed to enzyme-catalyzed metabolism. Relicts of such interactions might be preserved in modern metabolism.

## Figures and Tables

**Table 1:** Proposals for metabolic origins

	Origins environment	Source of					Defining catalysts	First cells
		Energy	C	e^−^	N	P		
A.	Ocean	Sugar-P	Sugar-P	Sugar-P	−	−	−	−
B.	Organo sulfidic	Thioesters	KEGG universal	H_2_	−	−	−	−
C.	Atmosphere	UV light	CO and CO_2_	H_2_O	NH_3_	−	−	−
D.	Surface impactor	UV light and impact	CN^−^	CN^−^ and H_2_S	CN^−^	Fe_3_P	−	−
E.	Terrestrial ponds	UV light	CN^−^	CN^−^ and ZnS	CN^−^	${\text{HPO}}_{4}^{2-}$	−	−
F.	Volcanos	FeS	CO_2_	FeS	NH_3_	${\text{HPO}}_{4}^{2-}$	FeS	−
G.	Serpentinizing hydrothermal vents	H_2_ and ${\text{HPO}}_{3}^{2-}$	CO_2_	H_2_	NH_3_	${\text{HPO}}_{3}^{2-}$	Ni^0^, Fe^0^, Co^0^, Pd^0^	Acetogens and methanogens

There are a number of theories for the origin of metabolism. They differ in their degree of detail and in the aspects of metabolism that they strive to explain. Most proposals for the origin of metabolism are tied in one way or another to a theory for the origin of life. References to the models listed are given in Box 1 (surface environment models A–E) and in Box 2 (subsurface environment models F and G).

**Table 2:** Native metal catalysis in primordial metabolism

Reaction	Native metal catalyst								Active site Ni or cofactor function replaced											Refs
	Ni^0^	Fe^0^	Ni_3_Fe	Ni_*n*_Co	Co^0^	FeCo	FeNICr	Pd^0^	NIFe Hyd	CODH	ACS	NAD (P)H	Mo(W) Co	h_4_f	h_4_mpt	F_420_	Cob	ATP	Other	
H_2_ activation																				
NAD^+^ + H_2_ → NADH	•	•			•				•											[64,65]
NMN + H_2_→ NMNH	•	•	•						•											[64]
Ferredoxin + H_2_ → ferredoxin^−^		•							•											[66]
Acetyl−CoA pathway, CO_2_ reduction																				
CO_2_ + H_2_ → HCOOH	•	•	•	• •	• •	•	•		•			•	•							[40,41,67–71]
CO_2_ + H_2_ → CH_3_COOH	•	•	•		•	•	•		•	•	•	•	•	•	•	•	•		TPP	[40,41,67–70]
CO_2_ + H_2_ → CH_3_COCOOH	•	•	•			•			•	•	•	•	•	•	•	•	•			[40,41,68–70]
CO_2_ + H_2_ → CH_3_OH	•	•	•	•	•	•	•		•	•	•	•	•	•	•					[41,72]
CO_2_ + H_2_ → C_2_H_5_OH				• •	• •	•			•	•	•	•	•	•	•	•	•			[71,73,74]
CO_2_ + H_2_ → CO				•		•	•		•	•										[71–74]
CO_2_ + H_2_ → CH_4_			•	•	•	•	•		•	•			•		•	•			F430	[41,71–74]
CO_2_ + H_2_ → C_2+_ hydrocarbons				•	•				•	•	•	•	•	•			•			[71,74]
CO_2_ + H_2_ → C_24_ hydrocarbons					•				•	•	•	•	•	•			•		CoA	[75],[76]^d^,[77]^e^
CO_2_ + H_2_O → HCOOH				•								•	•							[71]
CO_2_ + H_2_O → C_2_H_5_OH				•						•	•	•	•	•	•	•	•			[71]
Amino acid synthesis																				
Glyoxylate + NH_3_ + H_2_ → Gly	•								•											[78]
2-Oxoacid^a^ + NH_3_ + H_2_ → A, E, D, V, 1, L	•								•			•							PLP	[78]
Pyridoxal + NH_3_ + H_2_ → pyridoxamine	•								•			•								[79]
Intermediate metabolism																				
Pyruvate + glyoxylate → 9 rTCA acids^b^	•	•							•			•								[78,80,81]
Pyruvate + H_2_ + CO_2_ → citramalate			•						•			•							TPP	[68]
Oxaloacetate + H_2_ → succinate	•								•			•								[82]
Pyruvate + H_2_ → lactate	•								•			•								[79,83]
Pyruvate + H_2_O^c^ → lactate	•											•								[79],[83],[84]^f^
2-Oxoacids^c^ + H_2_O → 2-hydroxyacids	•											•								[83]
Fumarate + H_2_O^c^ succinate	•											•								[83]
Phosphoenol pyruvate 2-phosphoglycerate		•																		[85]
Phosphorylation																				
${\text{HPO}}_{3}^{2-}+\text{AMP}\to \text{ADP}+{\text{H}}_{2}$								•	•			•						•		[14,86]
${\text{HPO}}_{3}^{2-}+\;\text{serine}\;\to \;\text{phosphoserine}\;+{\text{H}}_{2}$								•	•									•		[14,86]
${\text{HPO}}_{3}^{2-}+\;\text{acetate}\;\to \;\text{acetyl}\;\text{phosphate}\;+{\text{H}}_{2}$								•	•									•		[86]
${\text{HPO}}_{3}^{2-}+\;\text{creatine}\;\to \;\text{phosphocreatine}\;+{\text{H}}_{2}$								•	•									•		[86]
${\text{HPO}}_{3}^{2-}+{\text{H}}_{2}\text{O}\to \;\text{pyrophosphate}\;+{\text{H}}_{2}$								•	•									•		[86]
${\text{HPO}}_{3}^{2-}+\;\text{ribose}\;\to \;\text{ribose}\;\text{phosphate}\;+{\text{H}}_{2}\;$								•	•									•		[86]
${\text{HPO}}_{3}^{2-}+\;\text{glucose}\;\to \;\text{glucose}\;\text{phosphate}\;+{\text{H}}_{2}$								•	•									•		[86]
${\text{HPO}}_{3}^{2-}+\;\text{glycerol}\;\to \;\text{glycerol}\;\text{phosphate}\;+{\text{H}}_{2}$								•	•									•		[86]
${\text{HPO}}_{3}^{2-}+\;\text{cytidine}\;\to \;\text{CMP}+{\text{H}}_{2}\;$								•	•									•		[86]

Circles indicate the formation of the products (rows) in the presence of the catalyst (columns). Reaction temperatures between 25 and 100°C in blue, and more than 150°C in red. Ni^0^, Pt^0^, Rh^0^, Ru^0^, and Ir^0^ also catalyze phosphite oxidation [15]. Reference [72–74] and some reactions in [71] report gas phase reactions; all others are liquid phase. Studies suggesting a role for ion gradients in CO_2_ fixation generate formate [87] and acetate [88] at yields several orders of magnitude lower than those obtained using shiny metals in pure water at 25–100°C, pH 8–12 without gradients. Gly: glycine; Hyd: hydrogenase; PLP: pyridoxal 5′-phosphate; rTCA: reverse tricarboxylic acid.

a Pyruvate, oxaloacetate, 2-oxovalerate, 2-oxoglutarate, 2-oxoisocaproate, and 2-oxo-3-methylvalerate.

b rTCA cycle reactions can also be catalyzed by lanthanides [89].

c Ni^0^ is the reductant; 2-oxoacids are 2-oxoglutarate, 2-oxoisocaproate, and 2-oxo-3-methylvalerate.

d Co in Co-bearing magnetite (Fe_3_O_4_) was likely reduced to Co^0^ during the reaction

e The reaction only worked in stainless steel reactors, not in glass reactors [77], see [72].

f Lactate to pyruvate oxidation is catalyzed by Ni^0^ in the presence of air [84].

**Table 3:** Laboratory reactions of phosphite catalyzed by Pd^0^

Reaction	Δ*G*_0_’ (kJ.mol^−1^)
${\text{HPO}}_{3}^{2-}+{\text{H}}_{2}\text{O}\to {\text{HPO}}_{4}^{2-}+{\text{H}}_{2}$	−46^a^
${\text{HPO}}_{3}^{2-}+\;\text{acetic}\;\text{acid}\;\;\to \;\text{acetyl}-\text{P}\;+{\text{H}}_{2}$	−3
${\text{HPO}}_{3}^{2-}+\;\text{creatine}\;\;\to \;\text{phosphocreatine}\;+{\text{H}}_{2}$	−3
${\text{HPO}}_{3}^{2-}+\text{AMP}\to \text{ADP}+{\text{H}}_{2}$	−15
$3{\text{HPO}}_{3}^{2-}+3{\text{H}}^{+}\to \;\text{trimetaphosphate}\;+3{\text{H}}_{2}$	−17^b^
${\text{HPO}}_{3}^{2-}+\;\text{ribose}\;\to \;\text{ribose}-5-\text{P}\;+{\text{H}}_{2}$	−13
${\text{HPO}}_{3}^{2-}+\;\text{glucose}\;\to \;\text{glucose}-\;\text{P}+{\text{H}}_{2}$	−15
$2{\text{HPO}}_{3}^{2-}+2{\text{H}}^{+}\to \;\text{pyrophosphate}\;+2{\text{H}}_{2}$	−15
${\text{HPO}}_{3}^{2-}+\;\text{cytidine}\;\to \;\text{cytidine}-5-\text{P}\;+\;{\text{H}}_{2}$	−31
${\text{HPO}}_{3}^{2-}+\;\text{serine}\;\;\to \;\text{phosphoserine}\;+{\text{H}}_{2}$	−34
${\text{HPO}}_{3}^{2-}+\;\text{glycerol}\;\to \;\text{glycerol}-\;\text{P}+{\text{H}}_{2}$	−37

Pyrophosphate: P_2_O_7_^4−^; trimetaphosphate: P_3_O_9_^3−^.

a Yang and Metcalf [111].

b Per metaphosphate bond, Meyerhof *et al*. [116]. Values of Δ*G*_0_′ for phosphorylation reactions were calculated from Δ*G*_0_′ of the phosphite oxidation reaction and Δ*G*_0_′ of the hydrolysis reaction using Hess’s law with values for free energy of hydrolysis from [15].

## Glossary

### Abbreviations

Acetyl phosphate: acyl phosphate with strong phosphoryl-transfer potential.
Acetyl–CoA pathway (Wood–Ljungdahl pathway): ancient autotrophic carbon fixation pathway shared by bacteria and archaea that reduces CO_2_ with H_2_ to form acetyl–CoA and pyruvate.
Acyl phosphates: compounds containing mixed anhydride bonds between carboxylate and phosphate groups.
Autocatalytic network: chemical reaction system in which the products generated by the network contribute, directly or indirectly, to sustaining or expanding the network itself.
Carbon monoxide dehydrogenase (CODH): ancient metalloenzyme of the acetyl–CoA pathway with active-site NiFeS clusters that catalyzes the reversible, ferredoxin-dependent interconversion of CO_2_ and CO.
Cobamides: cobalt-containing tetrapyrrole cofactors essential to the acetyl–CoA pathway, similar to cobalamin in structure but differing in the lower ligand.
Cofactors: nonprotein chemical compounds involved in enzyme activity, functioning as cosubstrates or as prosthetic groups, ionically or covalently bound to the enzyme.
Core metabolism: the set of approximately 420 biosynthetic reactions required to synthesize amino acids, nucleotides, and cofactors, from simple inorganic substrates such as CO_2_, H_2_, NH_3_, phosphate, sulfide, and water.
Cyanide: the simplest nitrile (a compound with a –C≡N moiety), is frequently invoked in prebiotic nucleotide synthesis.
Electrochemistry: study of electron-transfer processes involving an externally applied voltage and mineral or carbon electrodes.
FeS clusters: iron–sulfur cofactors of numerous enzymes involved in electron transfer and catalysis, with Fe atoms covalently bound to the protein via cysteine residues.
Hydride transfer: transfer of a hydride ion (H^−^) between molecules, commonly mediated by redox cofactors such as NAD(P)H or F_420_.
Hydrogenases: metalloenzymes with FeS, FeNiS, or Fe at their active site that catalyze the reversible oxidation of H_2_ to 2H^+^ and 2e^−^.
KEGG (Kyoto Encyclopedia of Genes and Genomes): database of biological pathways, genes, proteins, and metabolites for studying cellular processes and metabolism.
Microdroplet chemistry: chemical reactions occurring within charged microscopic droplets, such as those generated during MS.
Midpoint potential (*E*_0_′): measure of the tendency of a redox couple to donate or accept electrons under standard physiological conditions (pH 7 and 25°C).
Native metals: naturally occurring elemental metals, such as Fe^0^, Ni^0^, Co^0^, Pd^0^, Ni_3_Fe, and CoFe, that form under highly reducing geological conditions.
Palladium (Pd^0^): platinum-group metal that catalyzes phosphite-dependent phosphorylation reactions in water under mild conditions.
Phosphite $\left(HPO_{3}^{2-}\right)$: reduced phosphorus compound found in hydrothermal vents and used by many microbes.
Phosphoanhydride bond: high-energy phosphate bond linking phosphate groups in ATP, ADP, and related nucleotides.
Phosphorylation: addition or transfer of phosphate groups to molecules.
Platinum-group elements (PGEs): transition metals, including palladium, platinum, rhodium, ruthenium, iridium, and osmium.
Pyruvate synthase: enzyme catalyzing the reductive synthesis of pyruvate from acetyl–CoA, CO_2_, and reduced ferredoxin.
Reverse tricarboxylic acid (rTCA) cycle: autotrophic CO_2_ fixation pathway operating as the ferredoxin-dependent reductive counterpart of the oxidative TCA cycle.
Substrate-level phosphorylation: stoichiometric reaction between an organophosphate and ADP that produces ATP.
Thioesters: esters formed from organic acids and thiols. They have high group-transfer potential.

## Highlights

Last universal common ancestor’s metabolism encompassed approximately 400 reactions that convert H_2_, CO_2_, NH_3_, and inorganic phosphate into amino acids, nucleotides, and cofactors. Many of these reactions are catalyzed by shiny (native) metals, possible precursors of enzymes, and cofactors.

The acetyl-CoA pathway is the starting point of metabolism; it converts H_2_ and CO_2_ to pyruvate. Ni^0^ and Ni_3_Fe in water also convert H_2_ and CO_2_ to pyruvate as inorganic precursor of the pathway.

Transition metals in their shiny elemental (native) state—Fe^0^, Ni^0^, and Co^0^—occur naturally in serpentinizing systems. In the laboratory, they serve as broad specificity catalysts, replacing hydrogenases, the acetyl-CoA pathway, the tricarboxylic acid cycle, amino acid, and lipid synthesis.

Some serpentinizing systems harbor phosphite and native palladium. Pd^0^ catalyzes the aqueous phosphorylation of AMP, sugars, and acetate with phosphite, a novel source of energy at origins.
